# Genetic Analysis of CYP2C9 with Reference to Drug Response in Epilepsy Patients of Pakistan

**DOI:** 10.1155/2022/1451007

**Published:** 2022-01-29

**Authors:** Hafsa Maqbool, Tayyaba Saleem, Nadeem Sheikh, Aqsa Ashfaq

**Affiliations:** ^1^Institute of Zoology, University of the Punjab Lahore, Lahore, Pakistan; ^2^Department of Neurology, University Medical Center Göttingen, Robert-Koch-Straße 40, 37075 Göttingen, Germany

## Abstract

Epilepsy is a major global issue. Epilepsy patients are treated with AED (antiepileptic drugs). Interindividual variability in drug response has been documented in several studies. The resistance to drug response may be attributed to genetic polymorphism. The current study was undertaken to investigate the CYP2C9 gene polymorphism associated with antiepileptic drug (AED) resistance in the Pakistani population. The current study included 337 individuals including 100 control subjects, 110 drug-resistant subjects, and 127 drug responders. Genomic DNA was isolated from blood, and amplification of rs1799853 (430C > T) and rs1057910 was carried out by polymerase chain reaction. Genotypes of CYP2C9 SNPs were determined by Sanger's sequencing. Astounding results were observed in the current study that none of the well-known reported SNPs of CYP2C9 was found in our Pakistani cohorts. However, a novel missense variant (c.374G > A) was found only in drug-resistant patients of the current study. According to the *in silico* analysis performed by PolyPhen-2, it was observed that this nonsynonymous substitution is likely to be pathogenic. The results of our study demonstrated that rs1799853 and rs1057910 may be involved in drug resistance in the Pakistani population. However, some other variants on CYP2C9 may play a critical role in AED resistance that needs to be explored.

## 1. Introduction

Epilepsy is one of the highest incidence rate CNS illnesses, characterized by repeated seizures with a variety of symptomatology [1, 2]. Uncontrolled epileptic seizures reduce patients' quality of life by raising the risk of physical injury and having a detrimental impact on their physical and psychological well-being. Although the possible risk factors are well understood, it is still unclear why the efficacy of antiepileptic medicine administered to two people with the same kind of epilepsy or seizures may be so drastically different [3]. Genetic variables that alter the pharmacodynamic and pharmacokinetic characteristics of given medications are among the causes being considered. Currently, the thought under consideration by many scientists is that investigation of the mechanisms underlying drug resistance may lead to the development of novel treatment techniques. Recent research has increasingly shown that genetic variables, such as polymorphisms in the genes of microsomal enzymes involved in drug metabolism (CYP), have a role in the development of drug resistance in epilepsy [4–7]. The P450 cytochrome enzyme family is involved in the metabolism of the majority of medicines. The CYP2 family is in charge of the initial stage of exogenous particle metabolism. Many enzymes encoded by polymorphic genes are members of the CYP2 family. In terms of clinical significance, the enzymes CYP2C9, CYP2C19, and CYP2D6 are the most significant. CYP genetic variations were found in many cultures throughout the world, and the variations are responsible for the particular activity of drug-metabolizing enzymes [8]. Drugs, including antiepileptic medications, are biotransformed and eliminated by oxidation processes catalyzed by P450 cytochrome enzymes, as well as glucuronidation, in which CYP2C9 and CYP2C19 play a key role. The genetic polymorphisms of CYP2C9 and CYP2C19 impact the rate of drug metabolism, resulting in variable levels of sensitivity to prescribed therapeutic doses [9]. So far, 13 alleles of the CYP2C9 genes have been found, with CYP2C9*∗*2 and CYP2C9*∗*3 resulting from single-amino-acid substitutions in the CYP29C*∗*1 coding region, R144C, and I359L, respectively. It is linked to a drop in the phenytoin-metabolizing enzyme's activity [10]. The CYP2C9 polymorphism encompasses the presence of the three alleles CYP2C9*∗*1, CYP2C9*∗*2, and CYP2C9*∗*3, which are all characteristic of the Caucasian population. The prevalence frequencies of the *∗*2 (Arg144Cys) and *∗*3 (Ile359Leu) alleles in the Caucasian population are 8–12 percent and 3–8 percent, respectively. These are the alleles that cause a significant drop in isoenzyme activity. The proportion of *∗*2 and *∗*3 alleles is significantly lower in oriental and Afro-American groups, whereas various additional variants of the CYP2C9 isoenzyme producing gene (*∗*4, *∗*5, *∗*6) have been identified as being distinctive of these people [11]. Many medicines, including nonsteroidal anti-inflammatory medicines, antiepileptic medications, angiotensin II receptor antagonists, and antidiabetic and antithrombotic medicines, are biotransformed by the CYP2C9 isoenzyme. The genotype of a patient may aid in the selection and administration of antiepileptic medications. Literature evidence suggests a link between the CYP2C9 polymorphism and drug-resistant epilepsy; however, this link appears to be skewed by ethnicity [12–15]. The goal of the study was to identify a relationship between the SNP rs1799853 and rs1057910 of CYP2C9 gene in the Pakistani cohort.

## 2. Materials and Methods

### 2.1. Subjects

This study was approved by the Bioethics Committee of the Institute of Zoology University of Punjab, Lahore, Pakistan (Bioethics/133). Written informed consent was taken from every patient/guardian. All patients were recruited through the hospitals of Punjab, with standard, established clinical epilepsy diagnosis. Patients were eligible if they had drug-resistant or drug-responsive epilepsy, as described by the ILAE (International League Against Epilepsy) criteria and had been taking an AED for at least a year [16]. The patients were excluded if they have substantial psychiatric comorbidity, uncertain record of seizure frequency, experienced pseudoseizures, inconsistent AED therapy, drug addiction, and occurrence of neurodegenerative disorders. The study included 337subjects of which 110 were drug resistant, 127 were drug-responsive pediatric patients, and 100 were normal healthy individuals as a control group. Drug resistance was defined as consistent seizure frequency despite treatment with the maximum tolerance dose of two established AED. Drug responsiveness was defined as complete freedom from seizures for at least two years after treatment with AED. The participants of the study (patients/controls) were recruited from the same facility, shared similar ethnicity, and were unrelated. A structured questionnaire was used to collect demographic information as well as information on seizure forms and frequency, past medical history, AED history, and related family history. For DNA extraction and genotyping, a 3cc venous blood sample was taken. To ensure blind genotyping, a code was used for the identification of subject information and genotype data.

### 2.2. Genotyping

The genomic DNA was isolated from peripheral blood lymphocytes by the modified organic extraction method [17] and diluted to a final concentration of 2 0 ng/*μ*L with diethyl pyrocarbonate (DEPC) water. The selected SNPs were amplified using previously reported primers. The rs1799853 was amplified using CACTGGCTGAAAGAGCTAACAGAG as the forward primer and AGGAAGAGATTGAACGTGTGA as the reverse primer, while for rs1057910, GTGGGTGACCCTACTCCATATCAC was used as the forward primer and TTGACCTTCTCCCCACCAGCCTGC as the reverse primer [18]. The annealing temperature for rs1799853 and rs1057910 was 65°C and 60°C, respectively. Thermal cycling conditions were 5 min of initial denaturation at 95°C followed by 35 cycles of denaturation at 95°C for 45 s, annealing for 45 s at the abovementioned temperatures for each SNP, 45 s of initial extension at 72°C, and final extension at 72°C for 10 min. The confirmation of PCR products was made by resolving amplicon of 375 bp (rs1799853) and 130 bp (rs1057910) on 2% agarose gel. Genotyping was performed from a commercial source (1st BASE, Singapore).

### 2.3. *In Silico* Analysis

The sequences were visualized using BioEdit 7.0 software. BLAST was performed to compare all the sequences with the reference sequence (*Homo sapiens* Taxid 9606). Wild and mutant DNA sequences were translated and aligned by MEGA X software. Prediction of the clinical significance of the identified variants was made by PolyPhen-2software. Changes in the protein stability consequent to the identified mutations were accessed using mCSM software, and functional protein association analysis was performed by the online available tool STRING.

## 3. Results

**Figure 1:**
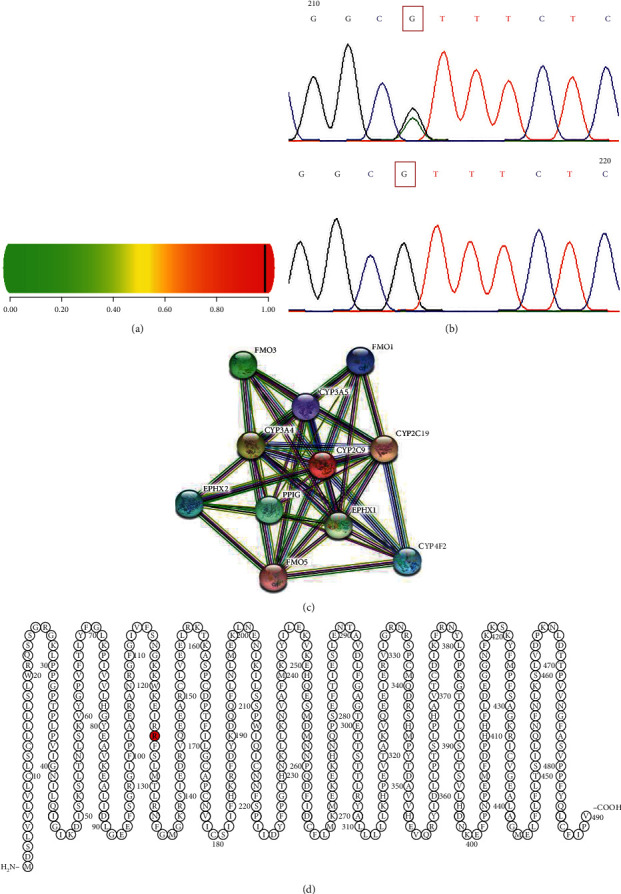
(a) The pathogenicity score for c.374G > A, (b) electropherogram of wild and heterozygous variant c.374G > A, (c) interaction of CYP2C9 with other functional nodes, and (d) predicted transmembrane topology with the highlighted variant position.

The mean age of the control group including 68 males and 32 females was 75 ± 21.89 months. The demographic attributes and seizure type for both groups, i.e., drug resistant and drug responsive, are presented in Table 1. None of the SNPs deviated from Hardy–Weinberg equilibrium in control subjects. We did not find any of the targeted mutations either in drug-responsive or drug-resistant epilepsy patients. We identified a novel missense variant at the c.374G > A position in CYP2C9 as shown in Figure 1(b). Genotype count for the identified SNP is given in Table 2.

### 3.1. *In Silico* Analysis

The alignment of mutated and reference sequence indicated a change in amino acid from arginine to histidine at the 125 position in CYP2C9 protein. The pathogenicity score for c.374G > A was 0.998, indicating this substitution to be likely pathogenic as presented in Figure 1(a). The position of the detected variant in polypeptide was determined by mCSM (Figure 1(d)). The predicted stability score (ΔΔG) was 0.44 kcal/mol. The interaction of the CYP2C9 gene with other function nodes is shown in Figure 1(c).

## 4. Discussion

Genetic and biomarker studies are still needed to identify epileptic patients at risk of developing medication resistance. Genetic variability has been demonstrated to contribute both in susceptibility and occurrence of disease and heterogeneity in therapy response [19]. Grover et al. reported about 20–95% of the clinical variation in drug effects and disposition linked to genetics. On average, AED monotherapy is successful in 60–70% of newly diagnosed epileptic patients, with a switch to another AED being successful in up to 50% of patients who fail the first AED treatment [20]. CYP2C9 is responsible for up to 90% of phenytoin hydroxylation [21]. The identification and characterization of genetic variants of the CYP2C9 gene might contribute to the optimization of antiepileptic drugs being administered. To the best of our knowledge, no studies have examined the relationship between CYP2C9 polymorphisms and drug-resistant epilepsy in Pakistani children. According to the global distribution analysis of CYP2C9 polymorphism, CYP2C9*∗*2 and CYP2C9*∗*3 variations are more common in European populations [22]. Asians have no CYP2C9*∗*2 mutation and have a very low CYP2C9*∗*3 frequency, ranging from 1.132 to 5.418. Both the CYP2C9*∗*2 (4 percent) and CYP2C9*∗*3 (2 percent) alleles were found to be rare among Asians and Africans [23, 24]. Two CYP2C9 alleles associated with impaired metabolism are found in 11 and 8% of Americans, but just 3 and 0.8 percent of Africans. The rs1799853 and rs1057910 polymorphisms of the CYP2C9 gene were evaluated genetically in order to determine their involvement in the development of drug-resistant epilepsy in children in the current investigation. These polymorphisms were chosen based on the previously cited research findings, which imply that they are linked to drug-resistant epilepsy. We could not find any of these well-known SNPs in the current study; however, a novel variant c.375G > A was found in the drug-resistant patients. This may confer the drug-resistant phenotype in epilepsy patients. According to a study conducted in a north Indian population, the CYP2C9 mutant allele has a protective effect against the development of AED resistance [21]. Previous research has found a substantial link between the CYP2C9 genotype and phenytoin maintenance dosage requirements [25]. van der Weide et al. discovered that phenytoin dose requirements are affected by CYP2C9 allelic variations; for patients with at least one mutant CYP2C9 allele, the mean phenytoin dosage required to produce a therapeutic blood concentration was roughly 37% lower than for wild-type people [25]. The mutant CYP2C9 alleles are linked to poor drug metabolism and function but on the other hand act as a barrier to the development of AED resistance. A report from Southern India showed the major role of CYP2C9 variants in phenytoin hydroxylation [26]. The polymorphic alleles change the activity of these isoenzymes, causing metabolism to be absent, diminished, or elevated. The findings of Lakhan et al. investigations revealed that CYP2C9 genetic variations have a substantial role in biasing epilepsy medication, demonstrating the importance of CYP2C9 mutants in preventing medication unresponsiveness in epileptic patients [27]. The rs1799853 polymorphism in the CYP2C9 gene has been linked to the incidence of T allele, which four times increases the likelihood of medication resistance in individuals with epilepsy [28]. The FDA (Food and drug Administration authority) has updated the clinical pharmacology part of the phenytoin label to include a warning concerning the risk of abnormally high levels in individuals who have specific CYP2C9 allelic variations [29]. Another study found a link between CYP2C9 polymorphisms and lower phenytoin metabolism and improved medication responsiveness. Nonresponders had a lower frequency of the heterozygous CYP2C9*∗*3 allele, according to the research by Lakhan et al. [27, 30]. In the current study, no differences in genotypes of drug-resistant, drug-responsive, and control groups were observed which may be attributed to various factors. Our study has some limitations. Firstly, the study's power may be insufficient as our sample size may not be large enough to establish a meaningful link. Perhaps in a larger investigation, it might be demonstrated. Secondly, some polymorphisms, such as those in the CYP2C9, CYP2C19, and UGT2B7 genes, are linked to the metabolism of some AEDs but not others. Our research did not aim to look at the link between polymorphisms and medication resistance in individuals on a specific AED. Additionally, drug resistance is a complicated trait that is influenced by several genes. The expression of multidrug transporters, in addition to drug-metabolizing enzymes, modulates phenytoin and carbamazepine disposition and may explain interindividual pharmacokinetic heterogeneity.

### 4.1. Conclusions

The patterns of genetic variations of the drug-metabolizing gene (CYP2C9) in different ethnicities translate into variation in drug response. Understanding CYP2C9 variability would improve rational drug usage and has public health implications. This study was designed to screen out the possible genetic variants of the CYP2C9 gene associated with AED resistance in Pakistani pediatric epilepsy patients. Our findings indicated an overall similar distribution of wild-type alleles in AED-resistant, AED-responsive, and control subjects. The wild-type alleles are reported to be associated with a higher rate of metabolism and early elimination of drug, thus requiring higher dose of AED to attain seizure freedom. The higher dose of AED is likely to be a contributing factor towards AED resistance. We also identified a novel variant. We analyzed only two SNPs of the CYP2C9 gene which were reported to be the hotspot for altering drug response. The pronouncement of CYP2C9 polymorphic contribution to drug response entails the screening of the whole gene on a larger scale in Pakistani pediatric epilepsy patients. The role of CYP2C9 polymorphism influence on drug response remains to be determined. Early detection of CYP2C9 polymorphism may help to predict and personalize the drug dose requirement. Further research is needed to validate the novel polymorphism found and those previously reported. It is noteworthy that multifactorial disorders such as epilepsy are influenced by a variety of factors that may be taken into account in the future.

## Figures and Tables

**Table 1:** Clinical and demographic attributes of drug-resistant and drug-responsive subjects.

Characteristics	Drug resistant, *N* = 110	Drug responders, *N* = 127
Age (months) (Mean + SD)	78 ± 26.25	71 ± 29.21
Gender		
Male	69	72
Female	41	55
Family history		
First-degree relatives	47	51
Second-degree relatives	11	21
Seizure type		
Myoclonic jerks	22	29
Generalized tonic-clonic seizures	35	49
Absence seizures	41	27
Atonic seizures	13	22
Infantile spasm	2	0
Tonic	19	21
Juvenile absence seizures	17	12

**Table 2:** CYP2C9 variant identified by sequencing.

Gene	Nucleotide change	Genotype count		
		Drug-resistant group	Drug-responders group	Control group
CYP2C9	c.374G > A	39/110	1/127	0/100

## Data Availability

The data used to support the findings of this study are available upon request from the corresponding author.
